# Supplementary material for: Evidence supported by Mendelian randomization: impact on inflammatory factors in knee osteoarthritis
Source: Front Med (Lausanne). 2024 May 28;11:1382836. doi: 10.3389/fmed.2024.1382836 (PMC11165061; doi:10.3389/fmed.2024.1382836)
Supplement: Supplementary file 1 [file Data_Sheet_1.ZIP › data/GCST90274815_5e6/815.leaveoneout.pdf]

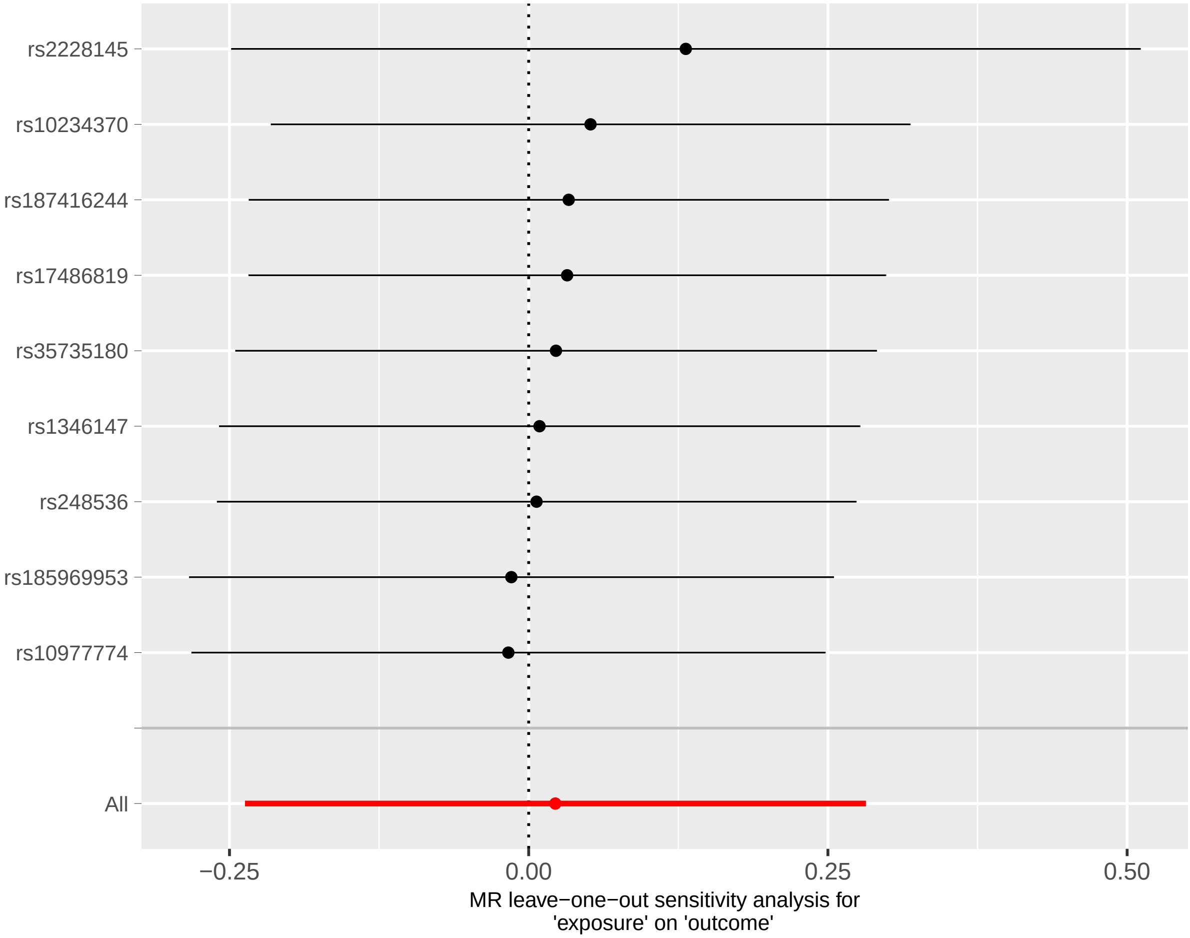
rs2228145
rs10234370
rs187416244
rs17486819
rs35735180
rs1346147
rs248536
rs185969953
rs10977774
All
−0.25
0.00
0.25
0.50
MR leave−one−out sensitivity analysis for
'exposure' on 'outcome'
